# Supplementary material for: H-Bonding Room Temperature Phosphorescence Materials via Facile Preparation for Water-Stimulated Photoluminescent Ink
Source: Molecules. 2022 Oct 1;27(19):6482. doi: 10.3390/molecules27196482 (PMC9571649; doi:10.3390/molecules27196482)
Supplement: Supplementary file 1 [file molecules-27-06482-s001.zip › molecules-1941988-supplementary.pdf]

H-bonding Self-assembly Room Temperature Phosphorescence Materials for Water-stimulated Photoluminescent Ink

Lingyun Lou,[a] Tianqi Xu,[a] Yuzhan Li,[a] Changli Zhang,[a] Bochun Wang,[a] Xusheng Zhang,[a] Hean Zhang,[a] Yuting Qiu,[a] Junyan Yang,[a] Dong Wang,[a] Hui Cao,[a] Wanli He[a] and Zhou Yang*[a]

[a] Department of Materials Physics and Chemistry, School of Materials Science and Engineering, University of Science and Technology Beijing, Xueyuan Road 30#, Haidian District, 100083, Beijing, People's Republic of China.
E-mail: yangz@ustb.edu.cn.

Supporting Information

Contents **Page**

Synthetic Procedures 2
Scheme S1 2
Theoretical calculations of DBAc-Cz and TBD 3
Scheme S2 3
Supplementary Data 4-9
Figure S1 4
Figure S2 5
Figure S3 6
Figure S4 7
Figure S5 8
Figure S6 9
Figure S7 9

Synthetic Procedures

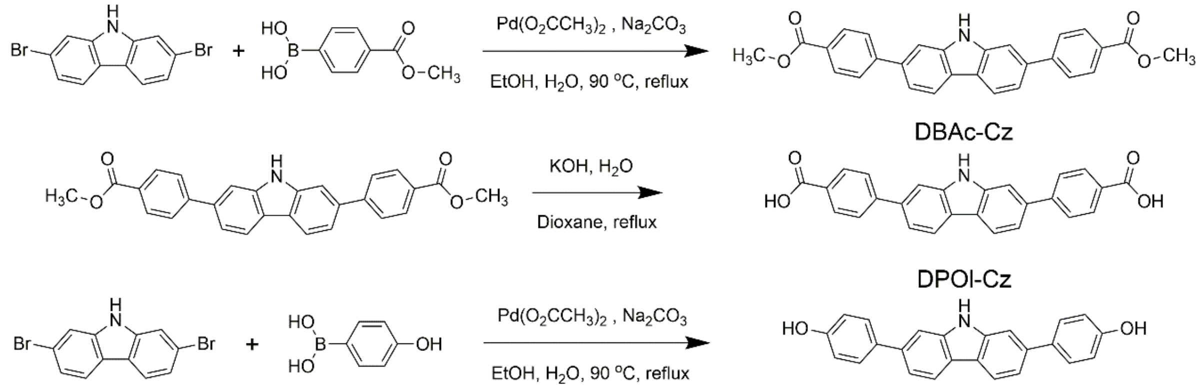

Scheme S1. Synthetic procedures of DBAc-Cz and DPOl-Cz.

Theoretical calculations of DBAc-Cz and TBD

DBAc-Cz and TBD is optimized optimized with dispersion corrected density functional theory (DFT) at the b3lyp/6-311/def2-SVP level. From the Kasha's rule, the fluorescence spectrum is usually related to the radiation transition of the first singlet excited state (S_1) while phosphorescence spectrum is related to the radiation transition of the first triplet excited state (T_1). In order to simulate the HOMO and LUMO orbitals, the fluorescence and phosphorescence emission spectrums of the two molecules, the molecular geometries at S1 and T1 excited states were then optimized at the b3lyp/6-311/def2-SVP level with the time-dependent density functional theory (TDDFT) method. All these DFT calculations above were performed using Gaussian 09W program suite.

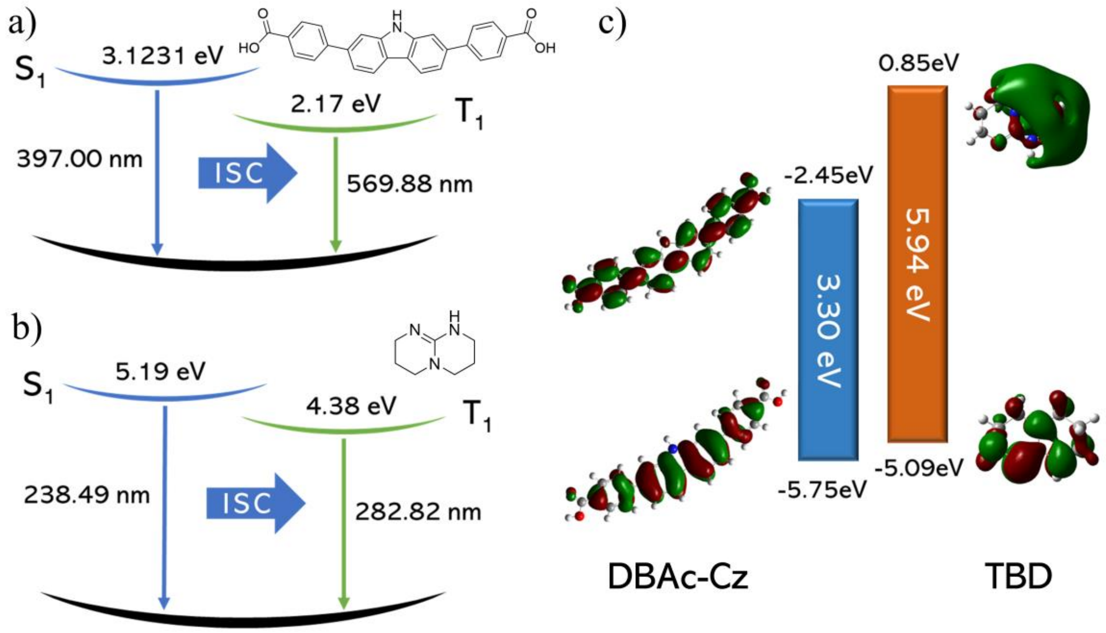

Scheme S2. Theoretical calculation results of DBAc-Cz and DPOl-Cz: a) DBAc-Cz's S_1 and T_1 states and fluorescence & phosphorescence emission wavelength. b) TBD's S_1 and T_1 states and fluorescence & phosphorescence emission wavelength. c) HOMOs and LUMOs of DBAc-Cz and TBD.

Supplementary Data

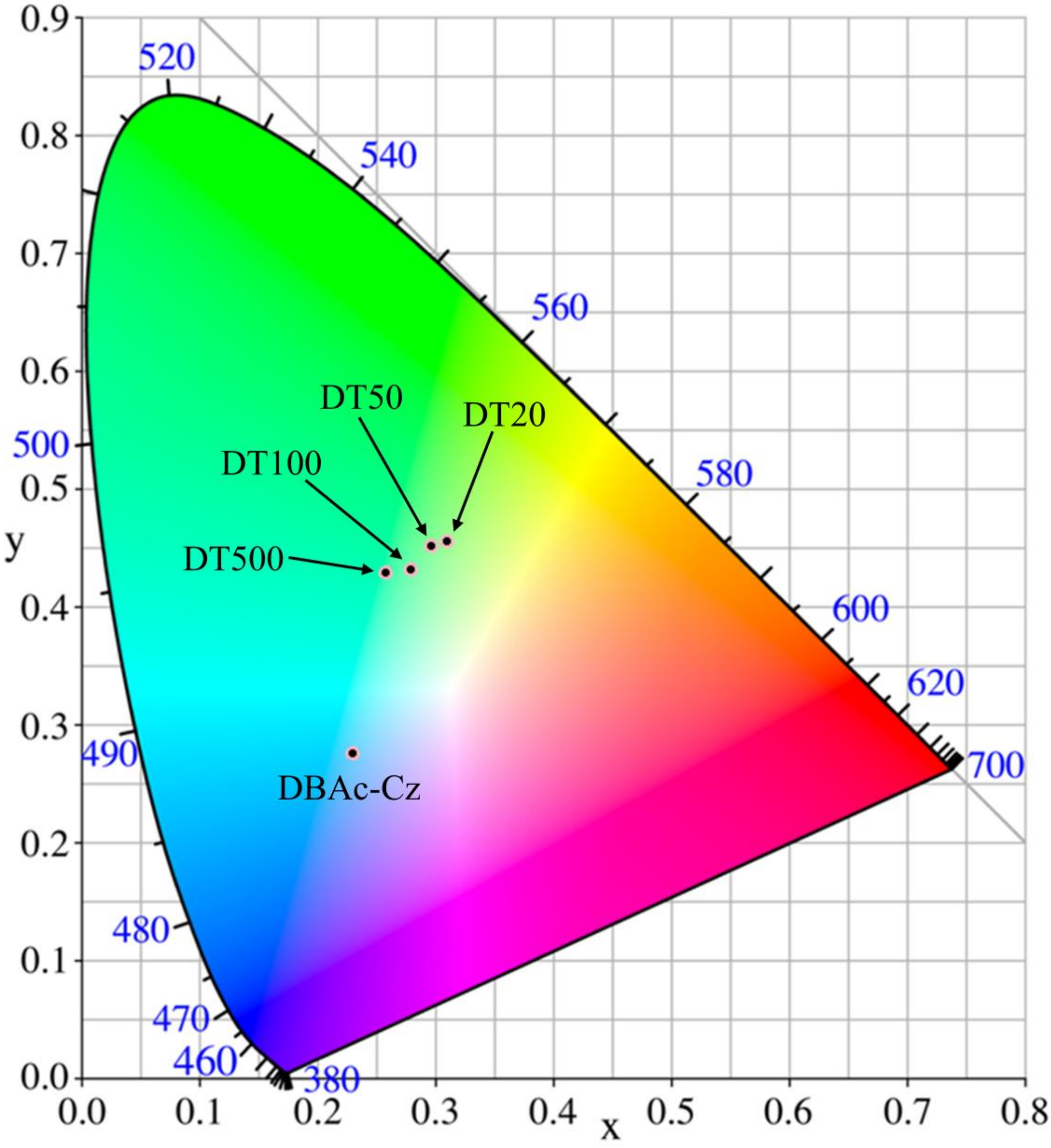

Figure S1. The Commission Internationale de L'Eclairage (CIE) chromaticity coordinate graph of DBAc-Cz and DTs fluorescence emission.

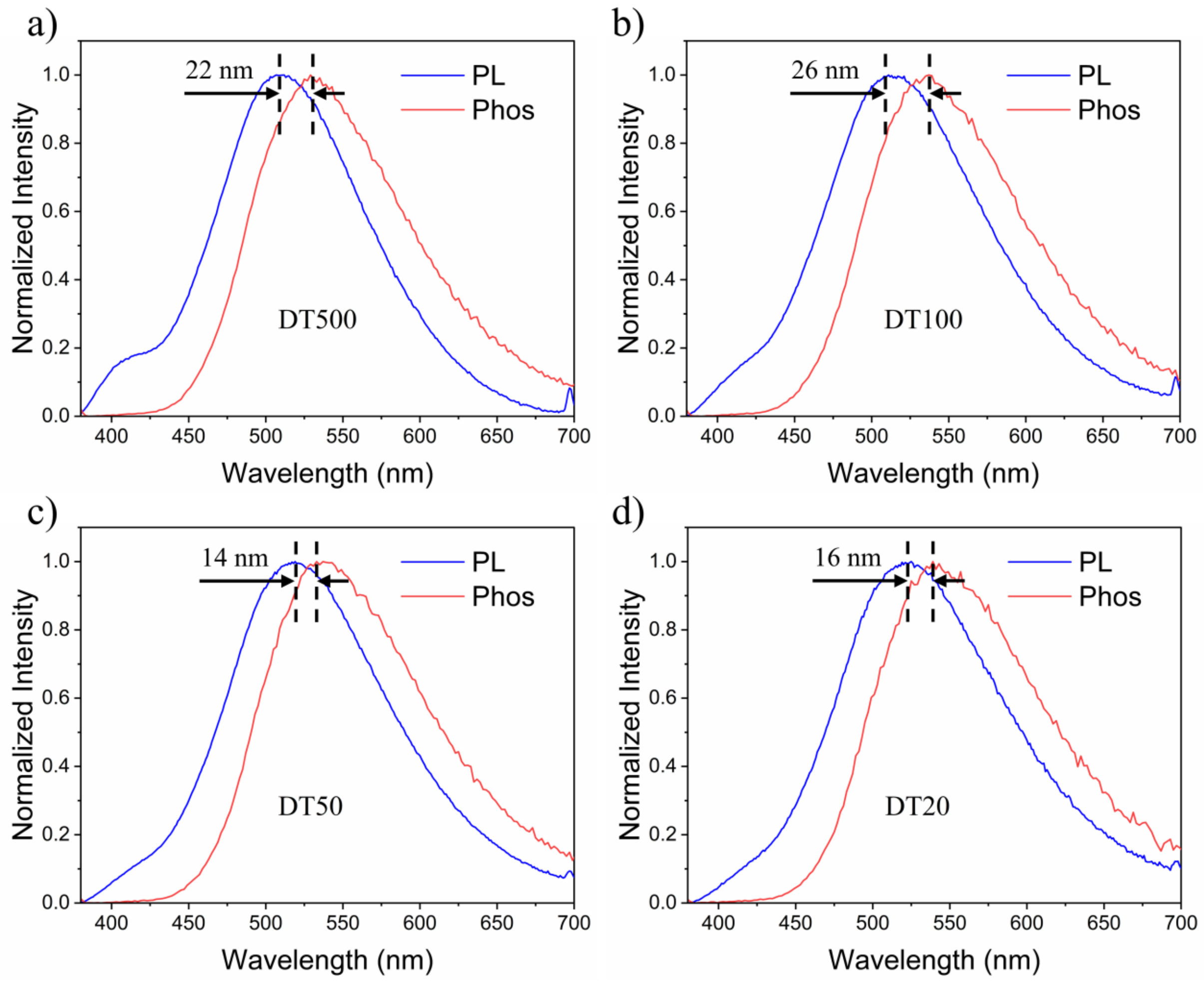

Figure S2. The comparison of steady-state PL spectra (blue line) and phosphorescence spectra (red line) of DTs.

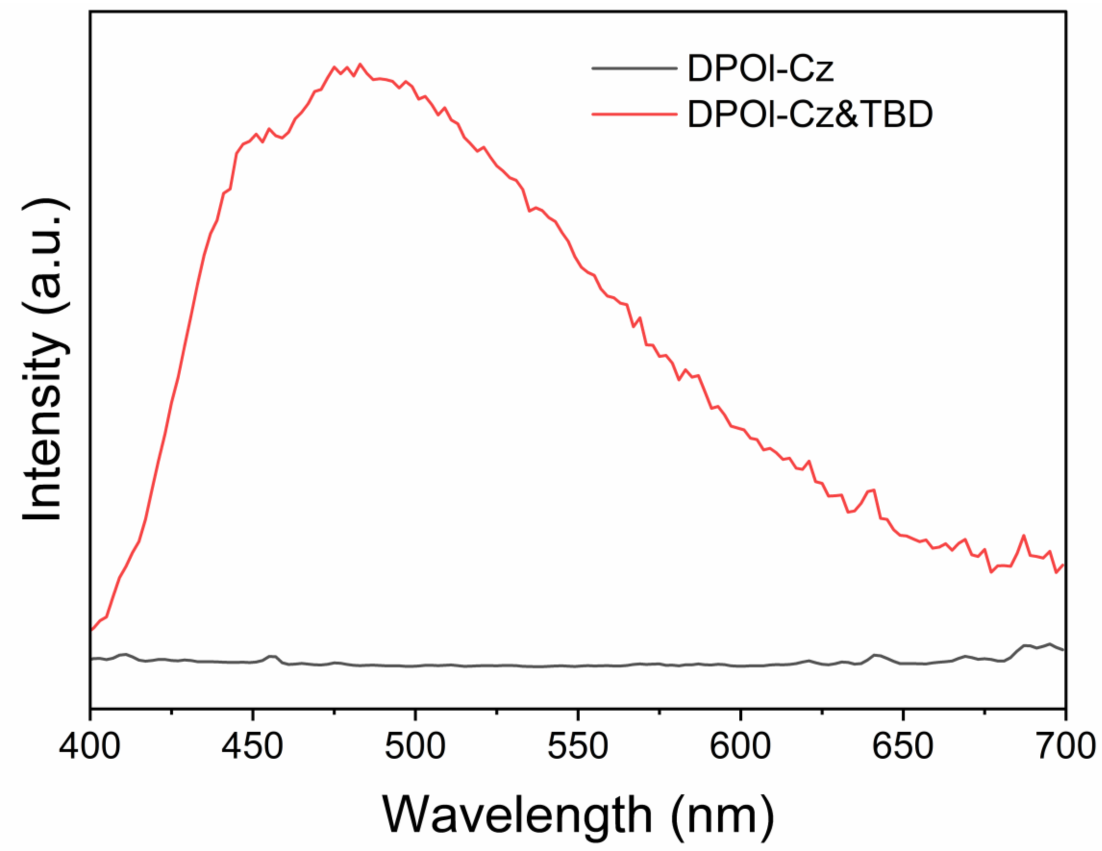

Figure S3. Photoluminescence spectra of DPOl-Cz and the grinded mixture of DPOl-Cz and TBD.

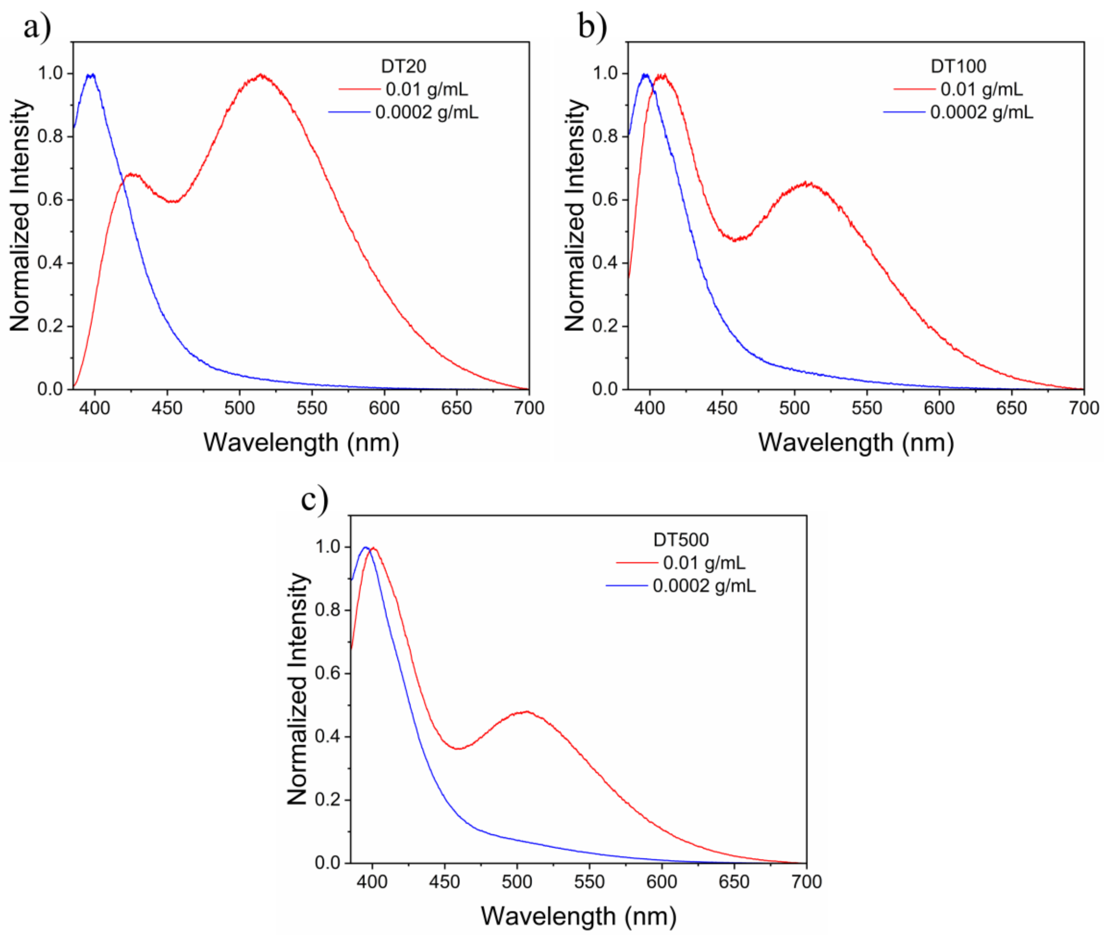

Figure S4. Fluorescence spectra of DCM solution of DT20, DT100, DT500 at different density.

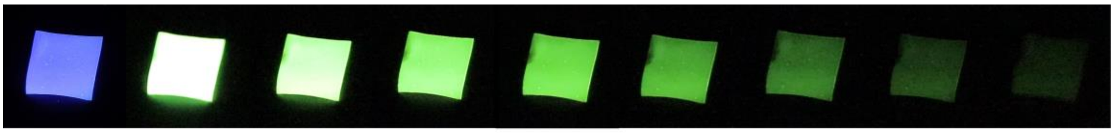

Figure S5. Luminescence photographs of PVA film doped with DBAc-Cz under 365 nm UV irradiation and at different time intervals after the removal of the ultraviolet lamp.

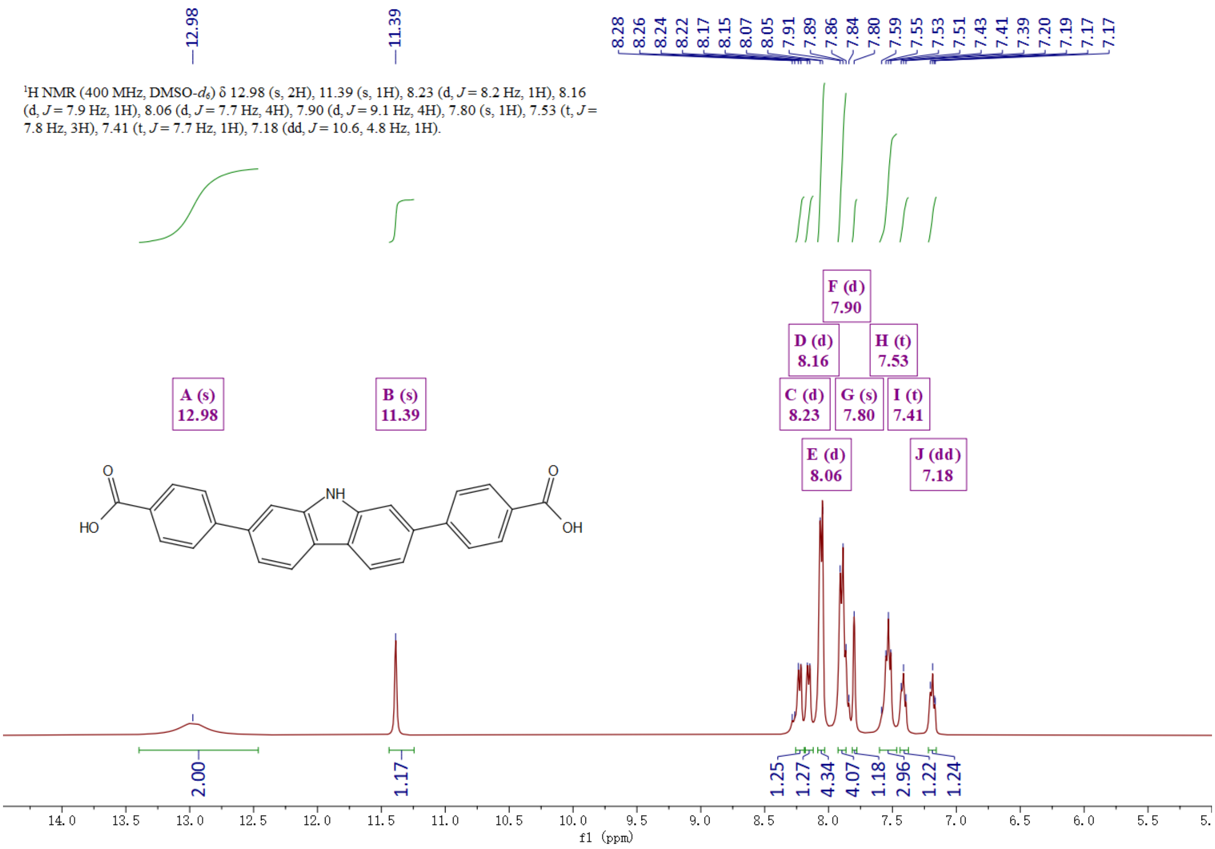

Figure S6. ^{1}H NMR spectrum of DBAc-Cz in DMSO-d_6.

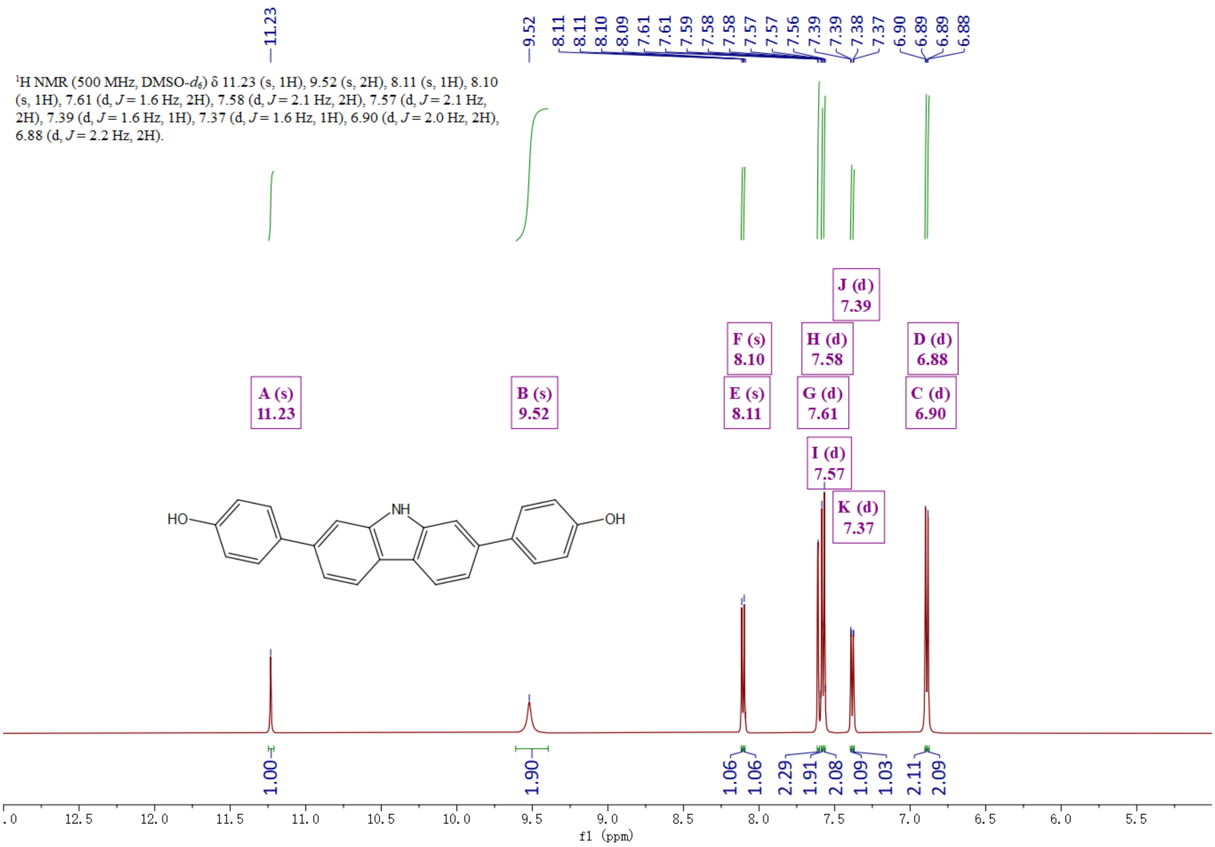

Figure S7. ^{1}H NMR spectrum of DPOl-Cz in DMSO-d_6.
